# Utilizing Melatonin to Alleviate Side Effects of Chemotherapy: A Potentially Good Partner for Treating Cancer with Ageing

**DOI:** 10.1155/2020/6841581

**Published:** 2020-05-21

**Authors:** Zhiqiang Ma, Liqun Xu, Dong Liu, Xiaoyan Zhang, Shouyin Di, Weimiao Li, Jiao Zhang, Russel J. Reiter, Jing Han, Xiaofei Li, Xiaolong Yan

**Affiliations:** ^1^Department of Thoracic Surgery, Tangdu Hospital, The Fourth Military Medical University, 1 Xinsi Road, Xi'an 710038, China; ^2^Department of Aerospace Medicine, The Fourth Military Medical University, Xi'an 710032, China; ^3^State Key Laboratory of Cardiovascular Disease, Fuwai Hospital, National Center for Cardiovascular Diseases, Chinese Academy of Medical Sciences, Peking Union Medical College, 167 Beilishi Road, Beijing 100037, China; ^4^Department of Cellular and Structural Biology, UT Health Science Center, 7703 Floyd Curl Drive, San Antonio, TX 78229, USA; ^5^Department of Ophthalmology, Tangdu Hospital, The Fourth Military Medical University, 1 Xinsi Road, Xi'an 710038, China

## Abstract

Persistent senescence seems to exert detrimental effects fostering ageing and age-related disorders, such as cancer. Chemotherapy is one of the most valuable treatments for cancer, but its clinical application is limited due to adverse side effects. Melatonin is a potent antioxidant and antiageing molecule, is nontoxic, and enhances the efficacy and reduces the side effects of chemotherapy. In this review, we first summarize the mitochondrial protective role of melatonin in the context of chemotherapeutic drug-induced toxicity. Thereafter, we tabulate the protective actions of melatonin against ageing and the harmful roles induced by chemotherapy and chemotherapeutic agents, including anthracyclines, alkylating agents, platinum, antimetabolites, mitotic inhibitors, and molecular-targeted agents. Finally, we discuss several novel directions for future research in this area. The information compiled in this review will provide a comprehensive reference for the protective activities of melatonin in the context of chemotherapy drug-induced toxicity and will contribute to the design of future studies and increase the potential of melatonin as a therapeutic agent.

## 1. Introduction

All organismal functions are affected by senescence, from the disorders of cellular protein production and alterations in the macroscopic characteristics of cells to the decline of organ or system functional efficiency, which may increase the development of age-related diseases such as cancer [1–4]. Chemotherapy is one of the main treatments for cancer patients [5, 6]. Chemotherapeutic agents are divided into several categories according to the factors of their effects, their chemical structures, and their relationships to other drugs [7]. The major categories of chemotherapeutic agents include anthracyclines (e.g., daunorubicin (DNR), doxorubicin (DOX), and epirubicin), alkylating agents (e.g., cyclophosphamide (CP), ifosfamide, melphalan, and busulfan), platinum (e.g., cisplatin and oxaliplatin), antimetabolites (e.g., 5-fluorouracil (5-FU), capecitabine, methotrexate (MTX), and gemcitabine), topoisomerase inhibitors (e.g., topotecan, irinotecan, etoposide, and teniposide), mitotic inhibitors (e.g., paclitaxel, docetaxel, vinblastine, and vincristine), and molecular-targeted agents (e.g., trastuzumab) [8, 9]. Despite advances in the development of effective chemotherapeutic drugs, their toxicity or adverse side effects to multiple organ systems and drug resistance have remained main barriers to their successful clinical application [7, 10]. For instance, alkylating agents and topoisomerase II inhibitors could increase the risk of secondary cancer (acute leukemia); anthracyclines, such as doxorubicin, can cause cardiotoxicity; and mitotic inhibitors may cause peripheral nerve damage [10].

Melatonin, a widely distributed and functionally diverse molecule, is also known as N-acetyl-5-methoxytryptamine [11–13]. In addition to influencing circadian rhythms, it modulates several molecular pathways related to antitumor effects, antiageing, anti-inflammation, sleep promotion, antivenom, body weight regulation, antidiabetic activity, and vasorelaxant and antifibrotic properties [14–18]. The roles of melatonin in alleviating chemotherapy drug-induced toxicity among the elderly have been widely considered, and a variety of new mechanisms have been confirmed [19–21]. Accumulated evidence suggests that melatonin enhances the efficacy and reduces the side effects of chemotherapy [22–24]. Pineal indoleamine has the double function of inhibiting cancer and protecting normal tissues, having low toxicity, being a highly effective free radical scavenger, and influencing mitochondrial homeostasis and functioning [25–27]. Furthermore, studies have demonstrated that melatonin was superior in preventing free radical destruction compared to other antioxidants, vitamin E, *β*-carotene, vitamin C, and garlic oil [28, 29]. Accordingly, the results generally showed that melatonin had a favorable therapeutic use in reducing chemotherapy drug-induced toxicity. However, the precise mechanisms of melatonin protection and the key cellular parameters of its influences still need to be clarified [30].

To study the protective actions of melatonin against chemotherapy drug-induced toxicity, herein we evaluated the available published documents regarding recent progress in this field. First, we present the evidence documenting the mitochondrial protective effect of melatonin on the toxicity of chemotherapy drugs. Second, we illustrate and discuss what is known about how melatonin protects against the detrimental roles of chemotherapy drug-induced toxicity while enhancing the efficacy of chemotherapeutic agents against tumor in various organs. Finally, we discuss several novel potential directions for future research in this field. Collectively, the information compiled here will provide a comprehensive reference for the actions of melatonin in protecting against chemotherapy drug-induced toxicity.

## 2. Correlation between Melatonin and Ageing

Accumulating studies indicate that melatonin is an antiageing agent that may retard the consequences of senescence [31]. Ageing results in circadian rhythm disorders, thus deregulating the melatonin synthesis [1]. The decrease of the melatonin peak in the elderly at night is consistent with the hypothesis that melatonin is related to senescence [32]. Melatonin mediated the important signaling pathways such as SIRT1 that is an ageing inhibitor whose upregulation may be associated with ageing [33]. Furthermore, senescence is an important pathogenic factor, which decreases the efficiency of the respiratory chain, increases electron leakage, generates free radicals, reduces the production of ATP, and finally leads to mitochondrial dysfunction [34], which can be ameliorated by melatonin. Additionally, melatonin may prolong life-span [35]. Chronic nighttime administration of melatonin significantly extended the life-span of mice and increased their immunocompetence [36, 37]. A recent report indicated that the synthesis of melatonin in mitochondria may be a key aspect of indole's role in ageing [14].

## 3. Senior Patients Are More Susceptible to the Side Effects of Chemotherapy

Senescence gradually restricts the functional reserve of multiple organ systems and affects the pharmacokinetics and pharmacodynamics of chemotherapy drugs, which may affect both the efficacy and the toxicity of chemotherapy [38]. The rate of functional complications of chemotherapy increases with age, including chronic cardiomyopathy, myelosuppression, nephrotoxicity, neutropenic infections, and chronic peripheral neuropathy [38]. Interestingly, there is a considerable overlap in the common biological changes that occur in normal ageing and chemotherapy treatment [39]. As the cause or consequence of mitochondrial dysfunction, oxidative stress is one of the main driving factors. Increasing reactive oxygen species (ROS) and reactive nitrogen species (RNS) levels occur in ageing and age-related diseases, which were also found in chemotherapy treatment [40]. Besides, ageing is related to cellular senescence, DNA damage, inflammation, mitochondrial dysfunction, and telomere length shortening, and chemotherapy is also similarly associated with all of these processes [39]. Pinder et al. demonstrated that there was a statistically significant increase in the risk of congestive heart failure in senior women who received anthracyclines [41]. The accumulation of cisplatin in the kidney of elderly mice was higher than that of young mice, which was highly correlated with the age-dependent sensitivity of cisplatin-induced nephrotoxicity. In addition, the changes in the inflammatory response and antioxidant signals of the elderly kidney led to the age-dependent susceptibility to kidney injury [42]. It was also indicated that senior patients are more susceptible to experience cognitive decline associated with chemotherapy for breast cancer than younger patients [43, 44]. Senescence appears to interact with cognitive reserve and increases the risk of cognitive decline after chemotherapy [43]. The above studies have shown that the biological processes of the ageing body's response to chemotherapy and degenerative changes overlap with each other, thus raising the hypothesis that ageing may increase the side effects of chemotherapy.

## 4. The Mitochondrial Protective Role of Melatonin in the Context of Chemotherapy Drug-Induced Toxicity in Ageing

**Figure 1:**
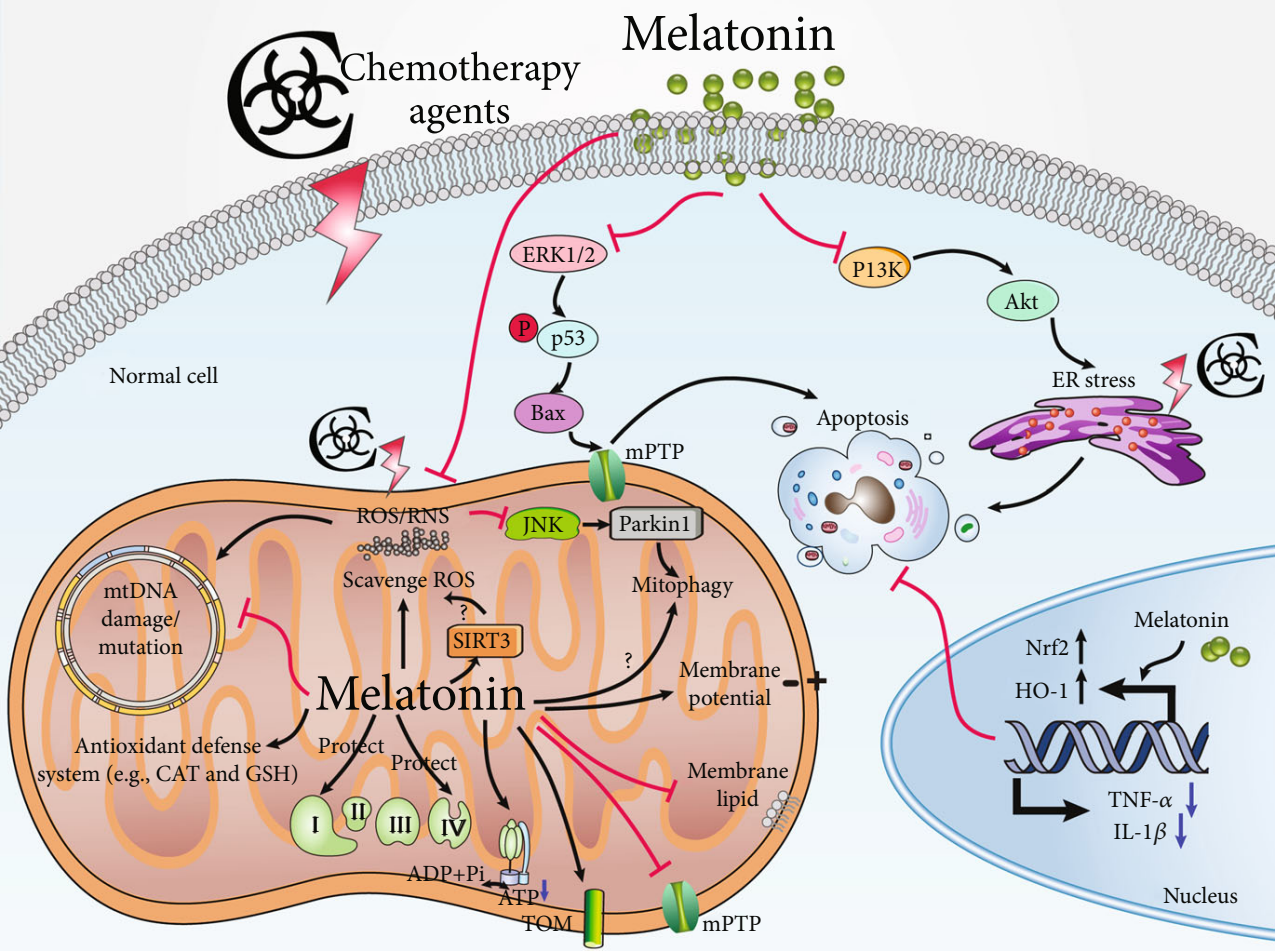
The mechanisms underlying cytoplasmic organellar dysfunction after chemotherapy and melatonin's protective effects under these conditions. Melatonin reverses chemotherapy-induced ER stress, as well as nucleus and mitochondrial dysfunction. In the mitochondrion, chemotherapy drugs lead to electron leakage and excessive free radical production. The ROS directly causes oxidative damage to the mitochondrial respiratory chain, further resulting in elevated electron leakage, free radical production, and ATP depletion. Moreover, ROS injures mitophagy, mtDNA, and the mitochondrial membrane structure (TOM complex reduction and mitochondrial membrane lipid peroxidation increases and elevates mPTP opening), leading to membrane potential loss and proapoptosis factor release. Apart from directly scavenging free radicals, melatonin protects against mtDNA damage/mutation, activates the antioxidant defense system, activates SIRT3 to scavenge ROS, and upregulates the TOM complex, the entry gate for the majority of precursor proteins that are imported into the mitochondria. However, the role of melatonin in mitophagy is less clear. Melatonin inhibits chemotherapy-induced stimulation of ERK1/2, followed via enhanced phosphorylation of p53 by the upregulation of genes such as Bax, thus resulting in mPTP opening. In the nucleus, melatonin upregulates Nrf2 and HO-1 expression and decreases TNF-*α* and IL-1*β* levels, thus contributing to cell protection. In the ER, melatonin reverses chemotherapy-induced ER stress via the inhibition of the PI3K/AKT pathway. As a consequence, melatonin protects diverse organs after chemotherapy. Abbreviations: Akt, protein kinase B; ATP, adenosine triphosphate; IL-1*β*, interleukin-1*β*; mPTP, mitochondrial permeability transition pore; ER, endoplasmic reticulum; ERK, extracellular regulated protein kinases; HO-1, heme oxygenase-1; JNK, c-Jun-N-terminal kinases; mtDNA, mitochondrial DNA; PI3K, phosphoinositide 3 kinase; ROS, reactive oxygen species; SIRT3, silent information regulator 3; SP, substance P; TOM, translocases in the outer membrane.

Mitochondrial dysfunction has been identified as an important event in chemotherapy-related toxicity in ageing [45, 46]. The mitochondrion is an organelle for ATP production and determines cell fate [30]. An important feature of mitochondria is that they are closely related to the senescence process, including generation of free radicals, production of ROS and RNS, amplification of damage caused by free radicals, and regulation of the apoptotic pathway due to interference with mitochondrial membrane potential and susceptibility to oxidative/nitrosative stress [1]. Furthermore, chemotherapy drugs often produce free radicals, which are a key cause of cell death [47]. ROS damage mitochondrial DNA (mtDNA), leading to the activation of the extrinsic apoptotic pathway (Figure 1) [48]. In addition, ROS interfere with calcium homeostasis and induce lipid peroxidation, reducing mitochondrial redox potential and opening the mitochondrial permeability transition pore (mPTP), thus resulting in membrane potential loss and cytochrome c release [48]. Excessive free radicals directly cause oxidative damage to the mitochondrial respiratory chain and metabolic enzymes, which further contribute to more electron leakage and free radical production (Figure 1) [49, 50]. Moreover, DOX reduces or inhibits the activity of the cellular antioxidant defense system that further leads to the oxidative stress [46, 51]. This leads to additional molecular damage thereby generating a vicious cycle that eventually leads to cell death [52, 53].

To reduce cell death, it is essential to break the vicious circle between free radical production and mitochondrial injury [14]. Melatonin, an effective free radical scavenger, is highly concentrated in mitochondria, which enhances its ability to resist mitochondrial oxidative damage [54, 55]. However, senescence can lead to the deterioration of circadian rhythmicity; thus, it causes disorders in melatonin synthesis [1, 56]. A certain amount of evidence has been accumulated showing that melatonin supplementation counteracts the exacerbating effects of senescence by inhibiting oxidative stress, mitochondrial dysfunction, and inflammation [1, 57]. Various studies have demonstrated that melatonin protected against mitochondrial dysfunction because of its direct free radical-scavenging activity and its indirect antioxidant properties [30, 58]. Melatonin effectively combats chemotherapy-mediated mitochondrial dysfunction by increasing the expression and activity of the mitochondrial respiration chain complexes (complexes I and IV), thereby increasing ATP production (Figure 1) [59]. When melatonin inhibits oxidative stress, lipid peroxidation is repressed and the mitochondrial membrane structure is protected [60]. It is also documented that melatonin regulates mitochondrial membrane permeability by modulating the translocases in the outer membrane (TOM) complex and mPTP activity (Figure 1) [61, 62]. In consequence, melatonin protects the mitochondrial membrane potential and inhibits the release of proapoptotic proteins [63, 64].

Compared with other antioxidants, the most attractive property of melatonin is that its metabolites also regulate the mitochondrial redox status by scavenging ROS and RNS and maintaining bioenergetic homeostasis and their antiapoptotic effects [30, 65]. Melatonin and its metabolites form a free radical-scavenging cascade, which makes melatonin highly effective even at low concentrations and can protect the organs against radical-induced damage continuously [66]. Furthermore, cyclic 3-hydroxymelatonin, a major metabolite of melatonin, protects mitochondrial cytochrome c against free radical-induced damage, and therefore it may inhibit apoptosis induced by oxidative cytochrome c release from mitochondria [67].

Additionally, compared with other antioxidants, melatonin has a protective effect on the heart without affecting DOX's antitumor activity, which is a unique characteristic of melatonin [68, 69]. Interestingly, some beneficial effects of melatonin on mitochondrial respiration are independent of its antioxidant activity but are related to its high redox potential [70]. This unique property allows melatonin to interact with the complexes of the electron transport chain, where it donates or accepts electrons thereby increasing electron flow in a way that other antioxidants do not [71]. Melatonin also exerts indirect protective effects through other pathways that do not involve radical scavenging. Melatonin enhances antioxidant defense systems by stimulating gene expression and the activity of antioxidants [59, 72] and improving the de novo synthesis of glutathione (GSH) by promoting the activity of gamma-glutamylcysteine synthetase [73].

Recent studies have revealed that melatonin inhibits the production of nitric oxide synthase (NOS) at the level of NOS gene transcription [74, 75]. Additionally, it was also shown that melatonin could selectively reduce mitochondria-induced NOS levels in the heart, thereby improving mitochondrial function in patients with sepsis [76]. In addition to the above effects, melatonin also maintains a healthy mitochondrial network by regulating mitochondrial biogenesis, dynamics, autophagy and mitophagy, mitochondrial fission and fusion, and its action on mitochondrial sirtuin activity [30, 77].

## 5. The Role of Melatonin in Anthracycline-Induced Organ Failure

The anthracyclines are the most widely used anticancer drugs in the treatment of human cancers, including their use in acute leukemia, Hodgkin's and non-Hodgkin's lymphoma, and breast cancer [78]. Like all other anticancer agents, anthracyclines are double-edged swords because they may result in the development of tumor cell resistance, and they are toxic to healthy tissues, especially the heart [78]. DOX and DNR are the original anthracyclines isolated from the pigment-producing bacterium *Streptomyces peucetius* in the 1960s. DOX differs from DNR by a single hydroxyl group, which has spurred researchers worldwide to identify five DOX/DNR analogs, one (idarubicin) of which is available in the United States [78]. A number of studies have indicated that DOX-induced cardiotoxicity is based on elevated oxidative stress via increasing ROS and lipid peroxidation, together with reducing the antioxidants and sulfhydryl groups [79, 80]. Compared with other organs, the heart has abundant mitochondria which are sources and targets of ROS, so that it is vulnerable to DOX-induced oxidative damage [45]. Moreover, the heart consumes more oxygen and has limited antioxidant defense systems compared with other tissues [81]. Thus, cardiomyocytes expressed low levels of catalase (CAT) and that antioxidant selenium-dependent glutathione- (GSH-) peroxidase-1 is inactivated when exposed to DOX, thereby reducing cytosolic antioxidant Cu-Zn superoxide dismutase [46, 51].

**Figure 2:**
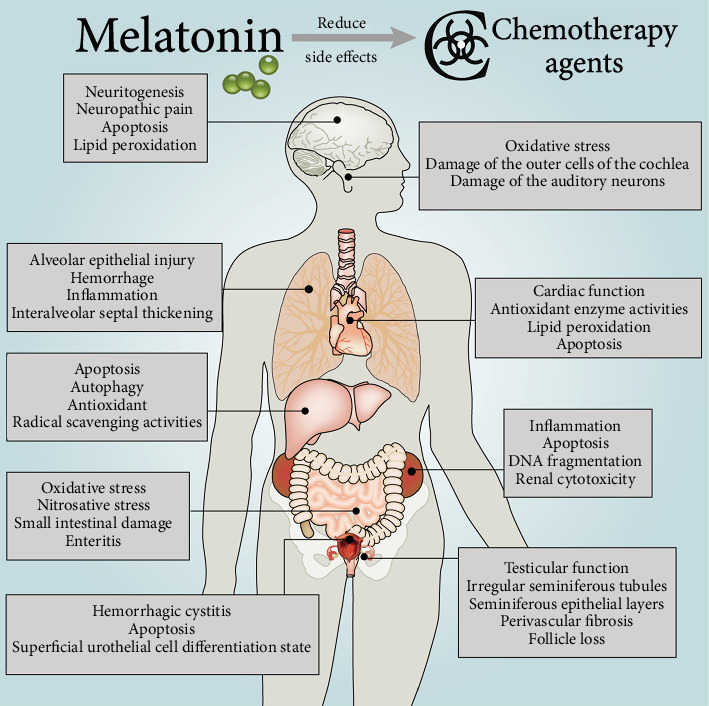
Protection of melatonin against chemotherapy drug-induced damage in various organs.

Although many approaches are designed to prevent or mitigate DOX toxicity, there are limits to the ability of these therapies to protect organs from injury, especially the heart. In contrast, the antioxidant melatonin has been effectively used to reduce cardiomyocyte damage [82, 83]. Melatonin plays a cardioprotective role against DOX-induced damage, including by elevating the ST segment and reducing the R-amplitude, decreasing the serum levels of cardiac injury markers, protecting antioxidant enzyme activity, reducing lipid peroxidation, and altering lipid profiles in the serum in rats (Table 1) [84]. Melatonin ameliorated oxidative stress by controlling iron and binding protein levels in DOX-treated rats [85]. Moreover, melatonin promotes the activity of protective antioxidative enzymes in myocardial cells subjected to the action of DOX. The protective effect is due to increased GSH levels and stimulation of CAT activity by melatonin in cardiomyocytes during DOX exposure (Table 1) [86]. Myocardial zinc accumulation may protect against DOX-induced oxidative stress, and melatonin inhibits the DOX-induced drop in plasma zinc levels, indicating that melatonin may have an action in maintaining plasma zinc levels [87]. Additionally, cardiac function was improved and lipid peroxidation was reduced after melatonin treatment, indicating that melatonin has a protective effect on DOX toxicity by attenuating lipid peroxidation (Figure 2) [88–90]. DOX binds to cardiolipin to form an irreversible complex; thus, it inhibits oxidative phosphorylation and prevents cardiolipin from acting as a cofactor of mitochondrial respiratory enzymes [91, 92]. Melatonin protects the mitochondria via inhibiting cardiolipin oxidation that would facilitate the mPTP, resulting in cell death [93]. Melatonin combined with DOX successfully inhibited DOX-induced apoptosis through AMPK-dependent mechanisms, indicating its potential as a cell death protectant in DOX chemotherapy [94, 95]. Another study reported that the protective effect of melatonin was due in part to inhibiting DOX-induced cardiomyocyte apoptosis by preventing DNA fragmentation (Figure 2) [30, 96, 97]. Furthermore, melatonin not only resists cardiotoxicity induced by DOX therapy, but it also enhances its antitumor activity more than vitamin E [98]. DOX causes serious injury when extravasated. Kesik et al. found that melatonin ameliorated DOX-induced skin necrosis in rats. Moreover, it could enhance the sensitivity of tumor to DOX *in vivo* [22, 99]. Melatonin administered in parallel with DNR reduced the proportion of apoptotic cardiomyocytes [100]. Epirubicin increased nitrosative stress only in heart tissue, and the cardioprotective effect of melatonin partially resulted from its suppression of epirubicin-induced nitrosative stress [101]. These results reveal that melatonin has a protective effect against epirubicin-induced cardiotoxicity [101].

## 6. The Role of Melatonin in Alkylating Agent-Induced Organ Failure

### 6.1. Cyclophosphamide

CP is most widely used an alkylating agent, and its antineoplastic and immunomodulating activities have been approved for early and advanced breast cancer [102]. CP alkylates DNA, forming DNA-DNA cross-links; thus, it inhibits DNA synthesis and causes cell death [103]. It was also shown that CP exerts its toxic effect via enhancing free radicals and other reactive oxygen species that cause lipid peroxidation and cell damage while melatonin provided an antioxidant defense with a highly chemoprophylactic effect on CP-induced cytotoxicity [104, 105]. Oxidative stress markers and the corresponding adaptability of the antioxidant defense system were increased after CP administration, indicating that oxidative stress plays a central role in CP-induced injury to the lung. Melatonin prevents CP-induced oxidative toxicity in pulmonary tissue [106]. Furthermore, by reducing bladder oxidative stress and blocking the production of nitric oxide synthase and peroxynitrite, melatonin upregulates heme oxygenase-1 (HO-1) and downregulates substance P (SP) expression, significantly improving bladder symptoms and lowering histological damage in CP-induced cystitis in rats (Table 1) [107, 108]. Another work reported that melatonin administration decreased bladder injury and apoptosis due to the upregulation of Nrf2 and nuclear transcription factor NF-*κ*B expression [109]. Moreover, melatonin cotreatment inhibited the development of hyperplastic urothelium, statistically significantly reduced cell proliferation and apoptosis index, and promoted the differentiation of superficial urothelial cells after CP treatment (Figure 2) [110]. CP caused spermatic tubule malformation, reduced the epithelium of spermatic tubules, and caused significant maturation stagnation and perivascular fibrosis [58]. Melatonin significantly improved the histopathologic appearance of a CP-damaged testis, indicating that the protective effect of melatonin on CP-induced testicular injury may be due to the antioxidant properties of indoleamine [58, 111]. Additionally, melatonin had potent antigenotoxic effects and suppressed chromosome aberrations against CP-induced toxicity in mice, which may relate to the scavenging of free radicals and elevated antioxidant status [112, 113]. Interestingly, the treatment of CP-induced hemorrhagic cystitis in rats with melatonin is characterized by the increased activity of the global autonomic nervous system (ANS) and a significant predominance of sympathetic tone, suggesting that melatonin may regulate autonomic activity through nonreceptor mechanisms [114].

### 6.2. Nitrogen Mustard

Nitrogen mustards, also known as DNA alkylating agents, are an important class of drugs for cancer treatment [115]. These anticancer drugs are used effectively in myelogenic leukemia; Hodgkin disease; lung, testicle, ovarian, and breast cancers; and several lymphomas [116]. Nonetheless, nitrogen mustards are highly reactive and lack selectivity; thus, they produce several adverse side effects [115]. Accumulation of inflammatory cells and increased proinflammatory cytokines, reactive oxygen species, nitric oxide, and peroxynitrite contribute to the pathogenesis of mustard-induced toxicity. Mechlorethamin (MEC), a nitrogen mustard, can result in alveolar epithelial injury, hemorrhage, inflammation, edema, and interalveolar septal thickening in lung tissues. Melatonin has anti-inflammatory properties, has the documented ability to alleviate mustard-induced toxicity, and acts as an iNOS inhibitor and a peroxynitrite scavenger in the lungs [117, 118]. Moreover, both inflammation and oxidative stress may be mechanisms in MEC-induced nephrotoxicity. TNF-*α* and IL-1*β* levels enhanced markedly with MEC application; melatonin ameliorated these increases and elevated NOx levels in kidney tissue. This supports the opinion that melatonin has anti-inflammatory and antioxidant properties [119]. Suppression of the mitochondrial pathway by melatonin significantly inhibited mustard-induced anoikis, indicating that suppression of caspase-dependent mitochondrial permeability transition preserves airway epithelial cells from mustard-induced apoptosis [120].

## 7. The Role of Melatonin in Platinum-Induced Organ Failure

### 7.1. Cisplatin

Cisplatin is widely used as a chemotherapeutic agent for the treatment of various malignant tumors in pediatric and adult patients, including non-small-cell lung cancer (NSCLC) and breast, testicular, and ovarian carcinomas [121]. However, the use of cisplatin is limited by its serious side effects such as nephrotoxicity and ototoxicity [122]. The decrease of antioxidant status induced by cisplatin results in the disorders of antioxidant defense against free radical damage [122]. Melatonin and its metabolites protect against cisplatin toxicity [123]. Melatonin directly scavenges the highly toxic hydroxyl radicals (•OH) and significantly attenuates renal cytotoxicity and DNA fragmentation induced by cisplatin [123, 124]. Melatonin treatment promoted the accumulation of Nrf2 and increased the expression of HO-1 in the cytosolic fraction, indicating that melatonin inhibits cisplatin-induced nephrotoxicity by activating the Nrf2/HO-1 pathway (Table 1) [122]. Cisplatin exhausts the dormant follicle pool in mouse ovaries via excessive activation of the primordial follicles without inducing follicular apoptosis. Pretreatment with melatonin effectively preserved the ovaries from cisplatin-induced injury, an effect mediated by the MT1 membrane melatonin receptor [125]. Furthermore, melatonin reduces cisplatin-induced follicle loss via blocking the phosphorylation of the PTEN/AKT/FOXO3a pathway (Figure 2) [126]. Cisplatin-treatment markedly impaired testicular function, but combined treatment with melatonin prevented the testicular toxicity in rats [111, 127]. Thus, melatonin is a potential therapeutic agent for protecting the reproductive system during chemotherapy.

The mechanism of the ototoxicity caused by cisplatin molecular damage is based on the generation of ROS, which interferes with the physiology of the organ of Corti. As an antioxidant and immune modulator, melatonin has been used to treat cisplatin ototoxicity using transtympanic local application in low doses [128, 129]. Melatonin attenuates cisplatin-induced cell death and reduced phosphorylated p53 apoptotic protein, cleaved caspase 3, and Bax levels, while enhancing the antiapoptotic Bcl-2 gene and protein expression [130]. It also reversed the effects of cisplatin through inhibiting the overexpression of mTOR and ERCC 1 and increasing the expression levels of Beclin-1 and microtubule-associated protein-light chain3-II, bringing about the development of intracellular autophagosomes [130]. These findings suggest that melatonin alleviated cisplatin-induced cell death in HepG2 cells by balancing the roles of apoptotic- and autophagy-related proteins [130].

Chemotherapy with cisplatin also has various vascular side effects. A recent report indicates that melatonin treatment protects the aorta during cisplatin-based chemotherapy [131]. Melatonin increased cisplatin-induced cytotoxicity and apoptosis in human lung adenocarcinoma cells [132]. Melatonin combined with chemotherapy had no effect on survival and adverse events in patients with advanced NSCLC, but showed a trend of improving health-related quality of life [133], which suggests that melatonin has the potential to treat NSCLC in combination with cisplatin.

### 7.2. Oxaliplatin

Oxaliplatin is a third-generation platinum compound which is active against colorectal growth, but its clinical application is limited attributed to peripheral neuropathy progression [134]. Mitochondrial dysfunction has been considered to be the main pathological mechanism of oxaliplatin-induced neurotoxicity, and the suppression of autophagy may also aggravate the death of neurons [135]. Melatonin has neuroprotective roles in oxaliplatin-induced peripheral neuropathy [135]. Moreover, melatonin protects against the oxaliplatin-induced pain and neuropathic deficits in rats [54, 135]. Melatonin suppressed the loss of mitochondrial membrane potential and Bcl-2/Bax ratio, as well as the release of sequestered cytochrome c, while promoting neuritogenesis in oxaliplatin-stimulated neuro-2a cells [135, 136]. Melatonin lowered oxaliplatin-induced mitochondrial lipid peroxidation levels and protein carbonyl content and regulated the changes of mitochondrial nonenzymatic and enzymatic antioxidants and complex respiratory enzymes [54]. It also improved oxaliplatin-mediated nitrooxidative stress to prevent nitrosylation of proteins and loss of antioxidant enzymes; thus, it ameliorates the function of the mitochondrial electron transport chain and maintains the biological energy of cells by increasing ATP levels [135]. The protective effects of melatonin are also partially due to the prevention of oxaliplatin-induced neuronal apoptosis via promoting the autophagy pathway of the peripheral and dorsal root ganglia (DRG) [135]. Melatonin was also found to inhibit proteolytic activation of caspase 3, inactivation of poly(ADP-ribose) polymerase, and DNA damage, thus allowing SH-SY5Y cells resistant to apoptotic cell death [136]. A recent study shows that melatonin reduces oxaliplatin-induced apoptosis via preventing GSH depletion and Mcl-1 downregulation in renal carcinoma Caki cells [137]. It has been documented that the neuroinflammatory response in the dorsal horn of the spinal cord is a key factor in oxaliplatin-induced pain. Melatonin has been reported to have anti-inflammatory and antiallodynia effects in preclinical and clinical pain studies [138].

## 8. The Role of Melatonin in Antimetabolite-Induced Organ Failure

MTX, a structural analogue of folic acid, is one of the most effective and potent anticancer drugs used for leukemia and other malignancies [139]. It is an important component in the treatment regime of acute lymphoblastic leukemia, lymphoma, osteosarcoma, and breast cancer, as well as in head and neck cancer [139]. However, its high toxicity, including gastrointestinal, renal, nervous, hepatic, and bone marrow toxicity, limits its use. The main toxic effects of MTX are intestinal injury and enterocolitis resulting in malabsorption and diarrhea [140], which are the major causes of morbidity in children and adults [141]. It is demonstrated that melatonin reduces MTX-induced oxidative stress and small intestinal damage in rats, indicating that supplementation with exogenous melatonin can significantly attenuate MTX-induced intestinal injury, and may be beneficial to the improvement of human enteritis induced by MTX (Figure 2) [141]. Moreover, a preclinical study reported that melatonin protected against MTX-induced small intestinal damage through reducing nitrosative stress, protein tyrosine nitration, and poly(ADP-ribose)-polymerase (PARP) activation (Table 1) [142]. Melatonin pretreatment attenuated MTX-induced oxidative stress, changed antioxidant enzyme activity, and improved myeloperoxidase activity, suggesting that melatonin may decrease renal damage via antioxidant and anti-inflammatory actions [143, 144]. Melatonin prevents MTX-induced hepatotoxicity through antioxidant- and radical-scavenging activities in male rats [145]. MTX treatment brings about enhanced malondialdehyde levels and myeloperoxidase activity and reduces GSH levels in the blood, liver, and kidney. These effects were reversed by melatonin, suggesting that melatonin may have a high therapeutic benefit when used with MTX [146].

## 9. The Role of Melatonin in Mitotic Inhibitor-Induced Organ Failure

Taxanes, including paclitaxel and docetaxel, are commonly used chemotherapeutic agents for a variety of malignancies [147, 148]. Paclitaxel has a wide range of anticancer effects. In the process of cell division, paclitaxel inhibits cell cycle and induces cell death by stabilizing microtubules and interfering with microtubule disassembly [149]. In addition to ameliorating disease-specific outcomes, taxanes also can cause considerable morbidity. The most common and particularly troublesome toxicity is taxane-induced peripheral neuropathy [150]. Melatonin plays a beneficial role in taxane-related neuropathy [151, 152]. Patients treated with melatonin during taxane chemotherapy had a lower incidence of neuropathy, suggesting that melatonin may be useful in preventing or reducing taxane-induced neuropathy and in maintaining quality of life [151]. Moreover, melatonin protected rats from paclitaxel-induced neuropathic pain and mitochondrial dysfunction *in vitro* (Figure 2) [153]. Mitochondrial dysfunction associated with oxidative stress in peripheral nerves has been considered as a potential mechanism [153]. The potential of melatonin to decrease mitochondrial injury and neuropathic pain due to paclitaxel has been documented [153].

## 10. The Role of Melatonin in Molecular-Targeted Agent-Induced Organ Failure

Trastuzumab, a humanized monoclonal antibody that can be used against the extracellular domain of human epidermal growth factor receptor 2 (HER2), is an important component of the adjuvant therapy and metastasis therapy for HER2-positive breast cancers. The herceptin adjuvant study reported that adjuvant trastuzumab treatment for 1 year improves disease-free survival and overall survival in patients with HER2-positive early breast cancer [154]. However, its side effects limit the use of adjuvant trastuzumab treatment, including cardiotoxicity, fever and chills, shortness of breath, muscle weakness, cutaneous rash, diarrhea, and headache [155]. Trastuzumab is an effective agent for the treatment of various neoplastic diseases. Oxidative stress markers and serum CK-MB levels were highly enhanced after treatment with trastuzumab; these changes were also reversed by melatonin treatment which resulted in near normal levels, which suggested that melatonin is effective in preventing trastuzumab-induced cardiotoxicity (Table 1) [156].

## 11. Melatonin Enhances the Efficacy of Chemotherapy Agents against Tumors

Mounting evidence indicates that melatonin exerts a variety of anticancer properties at different stages of tumor progression and metastasis [157–160]. Moreover, the combination of melatonin and chemotherapies has been reported to improve the effectiveness of anticancer drugs [23, 161]. Melatonin significantly enhanced the cytotoxicity of the chemotherapy drugs against cancer cells. Consistently, each of the chemotherapy drugs with melatonin increased the ratio of cells entering mitochondrial apoptosis due to ROS overproduction, mitochondrial membrane depolarization, and highly expanded DNA fragmentation [162, 163]. It was also reported that melatonin does not interfere with the action of DOX on cancer cells but actually enhances the action of the anticancer drug possibly by inhibiting the outflow of P-glycoprotein-mediated DOX from cancer cells [23]. Melatonin potentiates cisplatin-induced apoptosis and cell cycle arrest in human lung adenocarcinoma cells [132]. Costimulation of HeLa cells with cisplatin in the presence of melatonin further increased cellular apoptosis, improved the mitochondrial structure and function, and significantly increased caspase-9-dependent mitochondrial apoptosis [161]. Melatonin inactivated mitophagy via blocking c-Jun-N-terminal kinases (JNK)/Parkin, resulting in the suppression of antiapoptotic mitophagy, indicating that melatonin enhances human cervical cancer HeLa cell apoptosis induced by cisplatin through inhibiting the JNK/Parkin/mitophagy axis [161, 164]. Furthermore, melatonin substantially augmented the 5-FU-mediated inhibition of cell proliferation, colony formation, cell migration, and invasion of colon cancer cells [165]. It was shown that melatonin and 5-FU synergistically induced cell cycle arrest by activating the caspase/PARP-dependent apoptosis pathway [165]. Furthermore, melatonin exaggerated the antitumor role of 5-FU by inhibiting the phosphorylation of the phosphatidylinositol 3-kinase (PI3K)/AKT/iNOS signaling pathways or promoting the translocation of NF-*κ*B p50/p65 from the nuclei to the cytoplasm, abrogating their binding to the iNOS promoter; thus, it inhibits iNOS signaling [165, 166]. Additionally, melatonin intensified the antitumor actions of paclitaxel in the endoplasmic reticulum endometrial cancer cell line, which express MT1 melatonin receptors [167].

## 12. Potential Future Directions and Conclusions

Senescence is a process of gradual functional deterioration of physiological mechanisms as time goes on. About half of human deaths are linked to ageing-related chronic diseases, including neurological disorders, diabetes, cardiovascular diseases, and cancer [168, 169]. Mitochondrial dysfunction is the main driver of these processes, which occur in ageing and age-related disorders; they were also found in chemotherapy treatment [40]. Mitochondria are major production sites of free radicals and related toxic species [170]. Abnormal mitochondrial function, increased ROS production, damaged mitochondrial DNA, decreased respiratory complex activities, and augmented electron leakage and mPTP opening played key roles in the pathophysiology of chemotherapy agent-induced toxicity in ageing [30]. Consistent with the amphiphilic nature of melatonin, it easily crosses all biological barriers and gains access to all compartments of the cell, and it is highly concentrated in mitochondria, indicating its ability to resist mitochondrial oxidative damage [171, 172]. Melatonin was first implicated in modulating nuclear SIRT1 during the biological process of cancer [173, 174]. SIRT3 indirectly reduces cellular ROS to prevent the cardiac hypertrophic response [175]. Moreover, both MnSOD and catalase levels of SIRT3 transgenic mice were increased, implying that SIRT3 was partly responsible for the enhancement of the antioxidant defense mechanisms of the heart [175]. These evidences potentially indicate that melatonin may have the ability to regulate mitochondrial sirtuins during DOX-induced cardiotoxicity [176]. It is demonstrated that mtDNA lesions caused by ROS or ERK1/2 activation directly induced by DOX, followed by elevated phosphorylation of p53, upregulated genes such as Bax [177]. After melatonin pretreatment, ERK2, phosphorylated p38, HSP-70, phosphorylated p53, c-Jun, and other crucial stress protein levels returned to normal [178]. Additionally, excessive DNA damage and enhanced ROS production induced by DOX, resulting in PARP hyperactivation and energy depletion, promotes necroptosis [179, 180].

Melatonin also activates mitochondrial STAT3 via the SAFE pathway of decreasing myocardial IR damage [181–183]. In cultured neonatal rat cardiomyocytes and isolated rat hearts, melatonin precondition alleviates IR-induced mitochondrial oxidative damage by activating the JAK2/STAT3 signaling pathway, as well as enhances mitochondrial mitophagy via activating the AMPK-OPA1 signaling pathways [184–187]. Thus, melatonin may protect against chemotherapy agent-induced mitochondrial oxidative damage through similar pathways. Apart from melatonin as an effective free radical scavenger, it also maintains a healthy mitochondrial network by regulating mitochondrial biogenesis, dynamics, and mitophagy [188]. Melatonin has multiple mitochondrial benefits in Alzheimer's disease, by significantly reducing ROS-mediated mitochondrial fission, mitochondrial membrane potential depolarization, and mitochondrial tardiness, thereby stabilizing cardiolipin; collectively, these actions reduce enhanced mitochondria-mediated cell death [189].

It is well documented that DOX causes endoplasmic reticulum (ER) dilation, indicating that DOX may also affect ER function apart from actions in the mitochondrion [190]. ER is involved in protein folding, calcium homeostasis, and lipid biosynthesis [191]. ER stress refers to the accumulation of unfolded proteins induced by oxidative stress, ischemic injury, calcium homeostasis disorders, and/or enhanced expression of folded defective proteins [191]. DOX stimulates the ER transmembrane stress sensor, activating transcription factor 6, while suppressing X-box binding protein 1 expression, a gene downstream of activating transcription factor 6 [190]. The reduced expression of X-box binding protein 1 lowered the ER chaperone glucose-regulated protein 78 level that is crucial in adaptive responses to ER stress [190]. The results of this study revealed that DOX promoted the apoptosis response induced by ER stress without inducing the ER chaperone glucose-regulated protein 78, further elevating ER stress in the hearts (Figure 1). Moreover, doxorubicin activated caspase-12, an ER membrane-resident apoptotic molecule, which leads to cardiomyocyte apoptosis and cardiac dysfunction [190]. Melatonin reverses tunicamycin-induced ER stress by preventing the PI3K/AKT pathway, and it promotes cytotoxic response to DOX via enhancing C/EBP-homologous protein (CHOP) as well as decreasing surviving in human hepatocellular carcinoma cells [192, 193]. Therefore, in addition to protecting mitochondrial homeostasis, maintaining ER homeostasis may also be an important mechanism for melatonin to participate in antichemotherapeutic drug injury. This is an area where more intensive investigation is warranted.

A number of clinical studies have shown that melatonin treatment improves the efficacy and decreases the side roles of chemotherapy, prolongs survival time, and promotes quality of life for patients [82, 90, 194, 195]. The beneficial effects of melatonin administration are partially results from its direct free radical-scavenging activity and its indirect antioxidant properties [30]. Increasing reports on the role of melatonin in animal experiments and clinical trials will undoubtedly deepen our understanding of the protective and beneficial mechanisms of melatonin during chemotherapy. One clinical trial included 70 cancer patients (advanced NSCLC) who were treated with a combination of cisplatin plus etoposide or the chemotherapy drugs plus melatonin. On the basis of complete and partial tumor response rate, melatonin enhanced the effect of cisplatin plus etoposide, and improved the 1-year survival rate. Furthermore, the incidences of myelosuppression, neuropathy, and cachexia were significantly reduced, indicating that patients treated with melatonin had better tolerance to chemotherapy [23]. Another clinical trial treated a total of 250 patients with metastatic solid tumors who were given a variety of different chemotherapies alone or in combination with melatonin [23]. The objective tumor regression rate and the 1-year survival rate were again improved by melatonin cotreatment. Moreover, melatonin significantly alleviated the incidence of thrombocytopenia, neurotoxicity, cardiotoxicity, stomatitis, and asthenia [23]. The patients included in these studies were in the advanced stages of disease where any one treatment is likely to have little effect. Therefore, any benefit of melatonin therapy seems exceptional and applying melatonin therapy in the early stages of cancer seems reasonable, with the promise of greater benefits [23]. Although other clinical trials (NCT01557478, Phase 3; NCT02454855, Phase 3) related to melatonin alleviating the toxicity and improving the efficacy of chemotherapy have yet to be published, it seems necessary to use it more widely given the molecule's apparently beneficial properties and low toxicity. It is suggested that the therapeutic value of melatonin in chemotherapy-induced toxicity and its relationship with mitochondrial dysfunction in further double-blind placebo-controlled studies be evaluated; we anticipate a bounty of additional beneficial findings on the actions of melatonin cotreatment with chemotherapy agents in the next decade.

## Figures and Tables

**Table 1:** Protective effects and mechanisms of melatonin action against the side effect induced by chemotherapy agents.

Chemotherapy agents	Experimental studies	Drugs and doses	Administration route	Outcomes	Underlying mechanisms	References
Anthracyclines	NIH3T3 cells	DOX (2.6 *μ*M for 24 h) + melatonin (1 *μ*M for 24 h)		Countered apoptosis generated by DOX alone	AMPK-Ppar gamma-dependent mechanisms	[94]
	Male Wistar-Albino rats	DOX (18 mg/kg) + melatonin (10 mg/kg/day, 7 days)	Intraperitoneal	Protected the heart against DOX-induced cardiotoxicity	Melatonin treatment prevented the elevation of the ST segment and R amplitude, as well as the elevation of cardiac injury markers and lipid peroxidation, and it prevented the decrease of antioxidant enzyme activity	[84]
	Male Sprague-Dawley rats	DOX (10 mg/kg) + melatonin (15 mg/kg)	Intraperitoneal	Melatonin controlled oxidative stress and modulated iron, ferritin, and transferrin levels	Ameliorated oxidative stress by controlling iron and binding protein levels	[85]
	Buffalo strain rats	DOX (2.5 mg/kg) + melatonin (20 mg/kg)	Intraperitoneal	Melatonin stimulated the activity of protective antioxidative enzymes in myocardial cells of rats	Melatonin increased GSH levels and stimulated CAT activity	[86]
	Sprague-Dawley rats	DOX (15 mg/kg) + melatonin (84 mg/kg)	Intraperitoneal	Melatonin maintained the plasma zinc levels	Zinc accumulation protects against oxidative stress and melatonin inhibited the DOX-induced decrease in plasma zinc levels	[87]
	Male Wistar rats	DOX (7.5 mg/kg) + melatonin (6.0 mg/kg)	Intraperitoneal	Melatonin prevented DOX-induced lipid peroxidation in rat liver	Melatonin-induced gene expression changes	[87]
	Male Wistar rats	Melatonin (90 mg/kg)	Intraperitoneal	Cardiac function was improved and lipid peroxidation was decreased	Melatonin provides protection against DOX toxicity via an attenuation of lipid peroxidation	[88]
	Ehrlich ascite carcinoma-bearing mice	DOX (4 mg/kg/week, 2 weeks) + melatonin (5 mg/kg/day, 15 days)	Intraperitoneal	Melatonin protected against cardiotoxicity and enhanced its antitumor activity to a more significant extent than did vitamin E	The cardiac contents of total protein, GSH, and SOD were increased, while the cardiac content of MDA was decreased	[98]
	Male Wistar rats	Epirubicin (10 mg/kg) + melatonin (200 mg/kg)	Intraperitoneal	Melatonin protected against cardiotoxicity induced by epirubicin	Melatonin was partially attributed to the suppression of epirubicin-induced nitrozative stress	[101]

Alkylating agents	HBE cells	Yperite or with MEC + melatonin (100 *μ*M)		Melatonin prevented mustard-induced anoikis	Inhibition of caspase-dependent mitochondrial permeability transition	[120]
	NMRI mice	CP (200 mg/kg) + melatonin at different concentrations (2.5, 5, 10, and 20 mg/kg)	Intraperitoneal	Melatonin prevented CP-induced oxidative toxicity in mouse lung tissues	The activities of the antioxidant defense system, ROS scavenging, and free radical quenching were increased	[106]
	Female Wistar rats	CP (75 mg/kg) + melatonin (5 mg/kg)	Intraperitoneal	Melatonin significantly improved bladder symptoms and histological damage due to CP-induced cystitis	Diminishing bladder oxidative stress, blocking iNOS and peroxynitrite production, upregulating HO-1, and downregulating the expression of SP	[107]
	Male Sprague-Dawley rats	CP (150 mg/kg) + melatonin (10 mg/kg)	Intraperitoneal	Melatonin treatment reduced bladder damage and apoptosis	Upregulating Nrf2 and nuclear transcription factor NF-*κ*B expression	[109]
	Male ICR mice	CP (150 mg/kg) + melatonin (10 mg/kg)	Intraperitoneal	Melatonin cotreatment prevented the development of hyperplastic urothelium	Decreasing proliferation and apoptotic indices and causing the higher differentiation state of superficial urothelial cells	[110]
	Male Wistar albino rats	CP (100 mg/kg) + melatonin (10 mg/kg)	Intraperitoneal	Melatonin may reduce CP-induced testicular damage	The antioxidative properties of indoleamine existed in the chemical structure	[111]
	NMRI mice	CP (60 mg/kg) + melatonin (2.5, 5, 10, and 20 mg/kg)	Intraperitoneal	Melatonin has potent antigenotoxic effects and suppression of chromosome aberrations	Scavenging of free radicals and increased antioxidant status	[112]
	Albino Wistar rats	CP (75 mg/kg) + melatonin (40 or 100 mg/kg)	Intraperitoneal	Melatonin resulted in global ANS activity elevation, with a marked sympathetic tone predominance	Melatonin modulates autonomic activity via nonreceptor mechanisms	[114]
	Male Wistar rats	HN2 (0.5 mg/kg) + melatonin (20 mg/kg or 40 mg/kg)	Intraperitoneal	Melatonin reduced mustard-induced toxicity in the lungs	Melatonin restored oxidative and nitrosative stress markers in a dose-dependent manner	[117]
	Male Sprague-Dawley rats	MEC (3.5 mg/kg) + melatonin (100 mg/kg)	MEC via transdermal route and melatonin via intraperitoneal route	Melatonin has anti-inflammatory properties, as well as antioxidant properties	These increases and elevated NOx levels were ameliorated	[196]

Platinum	Hepatocellular carcinoma HepG2 cells	Melatonin (1 mM) + cisplatin (20 *μ*M)		Melatonin attenuated cisplatin-induced HepG2 cell death	Regulation of mTOR and ERCC1 expressions	[130]
	SK-LU-1 cell line	Cisplatin (11 and 4 *μ*M) + melatonin (1 or 2 mM)		Melatonin enhanced cisplatin-induced cytotoxicity and apoptosis	Elevating mitochondrial membrane depolarization, activating caspases-3/7, and inducing cell cycle arrest in the S phase	[132]
	SH-SY5Y cells	Melatonin (10 *μ*M, 50 *μ*M) + Oxa (100 *μ*M)		Melatonin protects against the oxaliplatin-induced pain and neuropathic deficits	Preventing the loss of mitochondrial membrane potential (*Ψ*m), inhibiting Bcl-2/Bax ratio and releasing sequestered cytochrome c, and promoting neuritogenesis	[136]
	HT-29 cells	Oxa (0-50 *μ*M) + melatonin (15 and 30 *μ*M)		Melatonin improved mitochondrial electron transport chain function and maintained cellular bioenergetics by improving the ATP levels	Ameliorating nitrooxidative stress and preventing nitrosylation of proteins and loss of antioxidant enzymes	[135]
	SH-SY5Y cells	Oxa (10 *μ*M, 50 *μ*M, and 100 *μ*M) + melatonin (10 *μ*M)		Melatonin attenuated oxaliplatin-induced apoptosis	Inhibition of GSH depletion and Mcl-1 downregulation	[136]
	Male Sprague Dawley rats	Cisplatin (7 mg/kg) + melatonin (5 mg/kg)	Intraperitoneal	Melatonin markedly reduced renal cytotoxicity and DNA fragmentation	Scavenge hydroxyl radical (•OH) directly	[123]
	Male Wistar rats	Melatonin (4 mg/kg, 10 days) + cisplatin (7 mg/kg)	Intraperitoneal	Melatonin suppressed cisplatin-induced nephrotoxicity	Increasing Nrf2 accumulation in the nuclear fraction and increasing the expression of HO-1	[122]
	Female Swiss mice	Melatonin (5, 10, or 20 mg/kg) + cisplatin (5 mg/kg)	Intraperitoneal	Melatonin effectively protected the ovaries against cisplatin-induced damage	The MT1 receptor and melatonin antioxidant effects	[125]
	Female CD-1 mice	Cisplatin (2 mg/kg) + melatonin (15 or 30 mg/kg)	Intraperitoneal	Melatonin attenuated cisplatin-induced follicle loss	Preventing the phosphorylation of PTEN/AKT/FOXO3a pathway	[126]
	Wistar rats	Melatonin (10 mg/kg) + Oxa (4 mg/kg)	Intraperitoneal	Melatonin ameliorated the mitochondrial lipid peroxidation levels and protein carbonyl content	Modulating altered nonenzymatic and enzymatic antioxidants and complex enzymes of mitochondria	[54]
	Male Sprague-Dawley rats	Melatonin (20 mg/kg + Oxa 5 mg/kg)	Intraperitoneal	Melatonin had anti-inflammatory and antiallodynia effects	Melatonin inhibited synthesis of inflammatory mediators	[138]

Antimetabolite	Male Wistar rats	Melatonin (20 and 40 mg/kg) + MTX (7 mg/kg)	Intraperitoneal	Melatonin reduced small intestinal damage and ameliorates MTX-induced enteritis	Attenuating oxidative stress and restoring the activities of the antioxidant enzymes	[141]
	Male Wistar rats	Melatonin (20 and 40 mg/kg) + MTX (7 mg/kg)	Intraperitoneal	Melatonin protected against MTX-induced small intestinal damage	Attenuation of nitrosative stress, protein tyrosine nitration, and PARP activation	[142]
	Male Wistar rats	MTX (7 mg/kg) + melatonin (40 mg/kg)	Intraperitoneal	Melatonin reduced renal damage via antioxidant and anti-inflammatory activities	Reduction of oxidative stress and alteration in the activity of antioxidant enzymes, as well as elevation in myeloperoxidase activity	[143]
	Male Sprague Dawley rats	MTX (13.4 mg/kg) + melatonin (10 mg/kg)	Intraperitoneal	Melatonin prevented MTX-induced hepatotoxicity	Through their antioxidant- and radical-scavenging activities	[145]

Mitotic inhibitors	Sprague Dawley rats, the 50B11 immortalized DRG neuronal stem cell line	Paclitaxel (100 *μ*M) + melatonin (1 *μ*M)	Intraperitoneal	Melatonin protected against neuropathic pain and limits paclitaxel-induced mitochondrial dysfunction in vitro	Limiting the development of mechanical hypersensitivity	[153]

Molecular-targeted agents	Male Sprague-Dawley rats	Trastuzumab (10 mg/kg) + melatonin (10 mg/kg, 2 days)	Intraperitoneal	Melatonin was effective in preventing trastuzumab-induced cardiotoxicity	Reversing oxidative stress markers	[156]

Abbreviations: CAT, catalase; CP, cyclophosphamide; DOX, doxorubicin; GSH, glutathione; MEC, mechlorethamin; MTX, methotrexate; Oxa, oxaliplatin; PARP, poly(ADP-ribose)-polymerase.
